# Spontaneous Retinal Waves Can Generate Long-Range Horizontal Connectivity in Visual Cortex

**DOI:** 10.1523/JNEUROSCI.0649-20.2020

**Published:** 2020-08-19

**Authors:** Jinwoo Kim, Min Song, Jaeson Jang, Se-Bum Paik

**Affiliations:** ^1^Department of Bio and Brain Engineering; ^2^Program of Brain and Cognitive Engineering, Korea Advanced Institute of Science and Technology, Daejeon 34141, Republic of Korea

**Keywords:** feedforward projection, iso-domain, long range connection, orientation map, retinal wave, visual cortex

## Abstract

In the primary visual cortex (V1) of higher mammals, long-range horizontal connections (LHCs) are observed to develop, linking iso-orientation domains of cortical tuning. It is unknown how this feature-specific wiring of circuitry develops before eye-opening. Here, we suggest that LHCs in V1 may originate from spatiotemporally structured feedforward activities generated from spontaneous retinal waves. Using model simulations based on the anatomy and observed activity patterns of the retina, we show that waves propagating in retinal mosaics can initialize the wiring of LHCs by coactivating neurons of similar tuning, whereas equivalent random activities cannot induce such organizations. Simulations showed that emerged LHCs can produce the patterned activities observed in V1, matching the topography of the underlying orientation map. The model can also reproduce feature-specific microcircuits in the salt-and-pepper organizations found in rodents. Our results imply that early peripheral activities contribute significantly to cortical development of functional circuits.

**SIGNIFICANCE STATEMENT** Long-range horizontal connections (LHCs) in the primary visual cortex (V1) are observed to emerge before the onset of visual experience, thereby selectively connecting iso-domains of orientation map. However, it is unknown how such feature-specific wirings develop before eye-opening. Here, we show that LHCs in V1 may originate from the feature-specific activation of cortical neurons by spontaneous retinal waves during early developmental stages. Our simulations of a visual cortex model show that feedforward activities from the retina initialize the spatial organization of activity patterns in V1, which induces visual feature-specific wirings in the V1 neurons. Our model also explains the origin of cortical microcircuits observed in rodents, suggesting that the proposed developmental mechanism is universally applicable to circuits of various mammalian species.

## Abstract (precis)

In the primary visual cortex (V1) of higher mammals, long-range horizontal connections (LHCs) are observed to develop, linking iso-orientation domains of cortical tuning. It is unknown how this feature-specific wiring of circuitry develops before eye-opening. Here, we suggest that LHCs in V1 may originate from spatiotemporally structured feedforward activities generated from spontaneous retinal waves.

## Introduction

**Figure 1.**
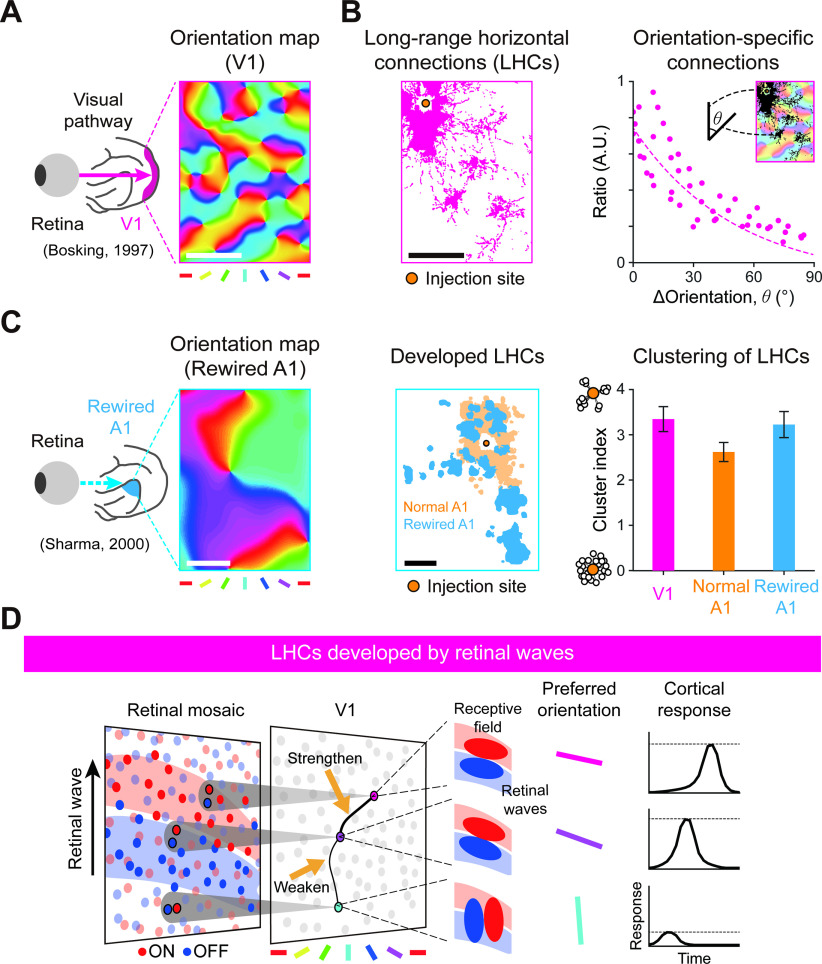
Feature-specific horizontal connections in V1 can be developed by retinal waves. ***A***, Orientation map in normal V1 (Bosking et al., 1997). Scale bar, 500 µm. ***B***, Distribution of horizontal connections over the cortical space of ***A***, and its density with respect to orientation difference. Scale bar, 500 µm. ***C***, Left, Orientation map in rewired A1 (Sharma et al., 2000). Middle, Distribution of horizontal connections in normal A1 and in rewired A1 showing that retinal afferent input induces the development of LHC-like long-range connections. Scale bar, 500 µm. Right, Cluster index of horizontal connections. ***D***, Illustration of the developmental model of orientation-specific connectivity by retinal waves. Following the statistical wiring model, local ON/OFF dipoles in an RGC mosaic are retinotopically wired to the V1, seeding cortical neuron anisotropic receptive fields and orientation tuning. The V1 contains a fully connected horizontal connection network, initialized with random synaptic strength. As a propagating retinal wave over the retinal mosaic coactivates cortical neurons with aligned ON/OFF subregions, connections between neurons with the same orientation preference are selectively enforced by the Hebbian learning rule.

In the primary visual cortex (V1) of higher mammals, neurons are observed to respond selectively to the orientations of visual stimuli, and their preferred orientations are organized into columnar orientation maps (Blasdel and Salama, 1986) (Fig. 1*A*). In addition, iso-domains of the same orientation preference in the map are linked together by long-range horizontal connections (LHCs) (Bosking et al., 1997) (Fig. 1*B*). The clustering of V1 by LHCs is observed prior to eye-opening (Weliky and Katz, 1994; Ruthazer and Stryker, 1996), suggesting that LHCs emerge prior to visual experience. Despite extensive studies on LHCs, it is still unknown what their functional role is (Gilbert and Wiesel, 1989; Stettler et al., 2002), and how they emerge before eye-opening.

In previous studies, it has been suggested that the feedforward afferents from the retina may play a critical role in the development of cortical circuitry (Callaway and Katz, 1991). Sharma et al. (2000) reported that rewiring retinal afferents to the primary auditory cortex (A1) in ferrets at early developmental stages results in development of orientation maps in A1 (Sharma et al., 2000) (Fig. 1*C*). Notably, LHCs, not observed in normal A1, are observed to emerge in the rewired A1. These results suggest that retinal afferents initiate development of the cortical orientation tuning and LHCs during early developmental stages. This scenario was further supported by observations that orientation tuning of cortical neurons originates from the local ON and OFF feedforward afferents (Jin et al., 2011; Kremkow et al., 2016). Moreover, the theoretical framework of the statistical wiring model indicates that the orientation tuning in V1 is constrained by the local structure of ON and OFF mosaics of retinal ganglion cells (RGCs) (Ringach, 2007; Paik and Ringach, 2011; Jang and Paik, 2017; Jang et al., 2020) (Fig. 1*D*). These findings inspired our hypothesis that the structure of retinal afferents may induce feature-specific wirings of LHCs in V1.

**Figure 2.**
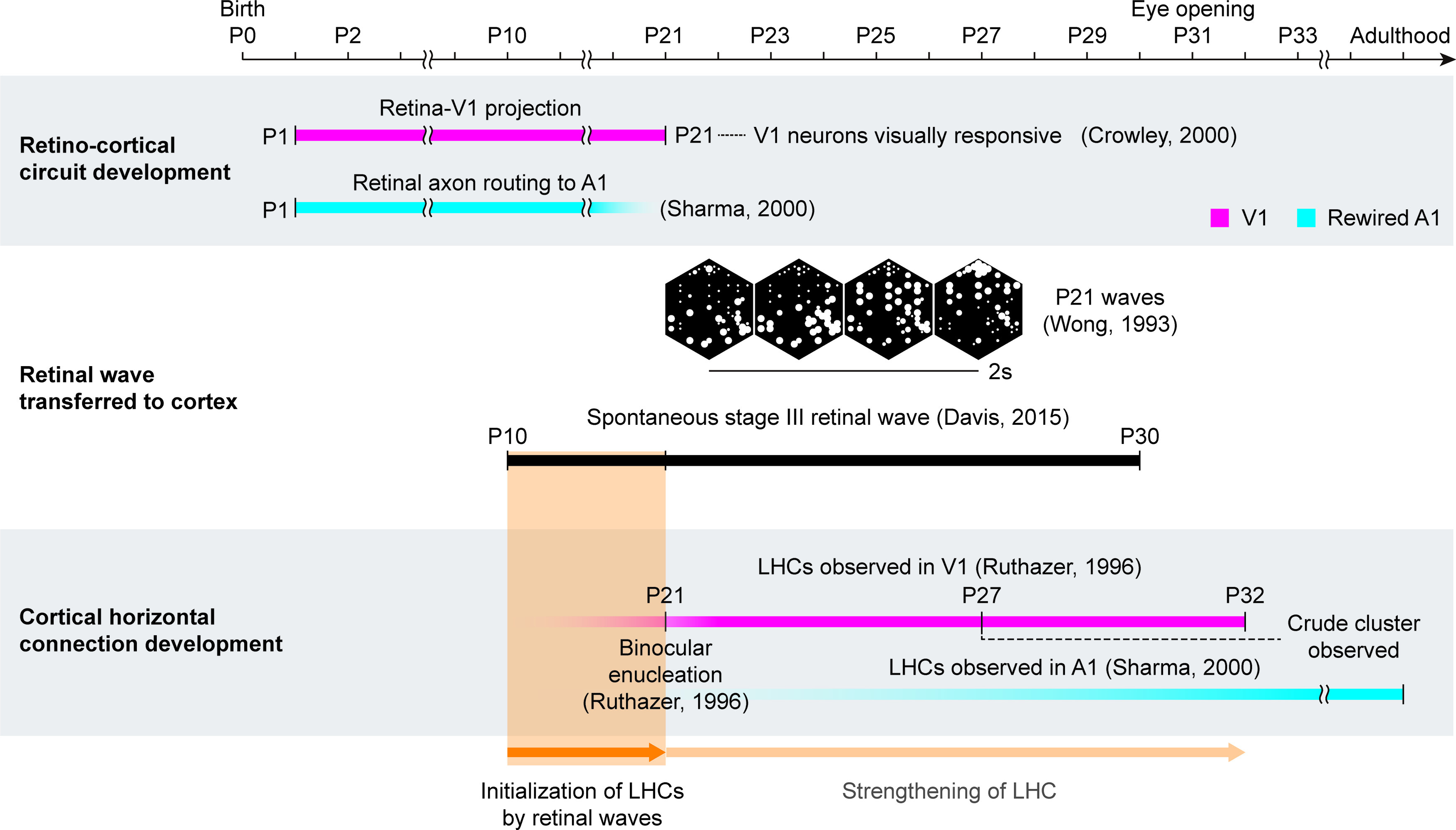
Development of long-range horizontal connections in V1 coincides with Stage III retinal waves. A developmental timeline illustrates a coincidence between the emergence of LHCs and the spontaneous Stage III retinal waves. A retinocortical pathway is already developing at P10 when geniculocortical afferents reach layer 4 (Sengpiel and Kind, 2002), and Stage III retinal waves are observed from P10 until eye-opening (P30) (Davis et al., 2015). This suggests that V1 neurons can be activated by these waves from P10. Clusters of LHCs are observed at P27, regardless of binocular enucleation at P21 (Ruthazer and Stryker, 1996). This implies that feature-specific LHCs can start emerging before then (orange shaded area).

Herein, from the model simulations of early visual pathways, we show that spontaneous retinal activity before eye-opening, which is spatiotemporally constrained by retinal mosaics circuitry, may selectively activate V1 neurons of similar orientation tuning and lead to development of LHCs via activity-dependent cortical plasticity. Our model is based on the observed data of spontaneous retinal activity (Meister et al., 1991; Wong et al., 1993) and their coincidence with the development of the visual circuits (Wong, 1999; Firth et al., 2005) (Fig. 2). It was reported that Stage II retinal activity induces development of the retinogeniculate and geniculocortical pathways (Ackman et al., 2012; Kirkby et al., 2013), and then the Stage III retinal activity that is observed until eye-opening (Liets et al., 2003; Akrouh and Kerschensteiner, 2013), coincides with the period of LHC development (Davis et al., 2015). Considering that the retinocortical projections are developed by the Stage II retinal activity, we hypothesized that the Stage III retinal activity is transmitted to the cortex and that this may play a crucial role in the development of LHCs.

From the simulations based on the anatomy of retinal circuits, we demonstrate that temporally asynchronous retinal waves from ON and OFF RGCs can drive V1 neurons selectively by theirorientation tuning, which drives feature-specific wirings of neurons with activity-dependent plasticity (Park et al., 2017; Lee et al., 2020) of horizontal wirings in the cortex. We also show that the developed LHCs can induce patterned cortical activities, matching the topography of underlying orientation maps as observed in ferrets before eye-opening (Smith et al., 2018). Finally, we demonstrate that LHCs can develop in the salt-and-pepper organization as observed in rodents (Van Hooser, 2005). These findings suggest that spontaneous retinal waves contribute significantly to organization of the functional architectures in the cortex during early developmental periods before sensory experience.

## Materials and Methods

### 

#### Model simulations

Model simulations were designed based on the statistical wiring model from the retina to V1 pathway (Ringach, 2007; Paik and Ringach, 2011; Song et al., 2018), and we performed the following: (1) generation of spontaneous retinal waves, (2) generation of orientation tuning of V1 neurons from statistical projections of RGC mosaics, (3) development of horizontal circuits in V1 by retinal waves, and (4) generation of spontaneous cortical activity from the developed V1 circuitry. Details of the algorithms, analysis methods, and parameters used in the simulations are presented in the following sections. All model simulations and data analyses were implemented and performed using MATLAB R2018a.

##### RGC mosaics

For the simulation of our retina-V1 model, we used ON/OFF-type RGC mosaics data from mammals. Simulations shown in the main results are based on cell body mosaics of the cat (Zhan and Troy, 2000) and mouse (Bleckert et al., 2014). We also provided simulation results based on monkey receptive field mosaics (Gauthier et al., 2009). Considering that differences in cortical tuning organization, modeling, and simulation schemes differ between higher mammals (cat, monkey) and rodents (mouse), from here, we describe the framework for simulating higher mammalian visual cortex and later describe specifications for the rodent cortex model.

For an RGC mosaic, we denote positional vectors of ON/OFF-type cells as $p_{i}^{ON},p_{j}^{OFF}$, where *i* and *j* denote the cell index. We also measured the density of OFF RGC, and from that, we defined a representative spacing $d_{OFF}$ so that an ideal hexagonal RGC lattice with spacing $d_{OFF}$ would have the same cell density as the data OFF mosaic. We computed $d_{ON}$ in the same manner.

##### Extending a data mosaic

To simulate spontaneous retinal waves before eye-opening, we modeled the retinal circuitry as a coupled network between ON RGC, OFF RGC, and amacrine cells (ACs) (Akrouh and Kerschensteiner, 2013). Because the data mosaic represents a limited region of the retina (several hundred micrometers) and lacks AC positional information, we first augmented the data RGC mosaic with surround RGC padding and an AC lattice. Added cells were only used to aid wave propagation, and we did not consider them in further simulations.

First, to provide a retinal region sufficient for the wave to propagate, we padded the data RGC mosaic with synthesized hexagonal lattices of ON and OFF RGCs, each with spacing $d_{ON}$ and $d_{OFF}$. With this spacing, a synthesized lattice has the same cell density as the data mosaic. We gave the padded lattice a circular boundary of radius 3000 µm to reduce potential propagation direction bias.

Then, a hexagonal AC lattice of (ON density + OFF density) was synthesized and overlaid on the augmented RGC mosaic. The resulting extended mosaic is an overlap of large ON/OFF/AC lattices with circular boundary, and we denote positional vectors of each type of cell as ${\hat{p}}_{i}^{ON},{\hat{p}}_{j}^{OFF},{\hat{p}}_{k}^{AC}$, where *i*, *j*, and *k* denote the cell index. When referring to an extended mosaic, we use hat notation $\hat{p}$ instead of the term *p* used for a data mosaic.

##### Network model generating retinal waves

In previous studies, it was reported that at least five cell types (including cone bipolar cells, ACs, and RGCs) and several different transduction mechanisms contribute to retinal waves. Based on the circuit mechanism reported by Akrouh and Kerschensteiner (2013) and the Stage II wave model proposed by Butts et al. (1999), we designed a simplified retinal circuitry model involving ON/OFF/AC cells, where three types of local connectivity drove retinal wave propagation: ON→ON excitatory, ON→AC excitatory, and AC→OFF inhibitory (Butts et al., 1999; Akrouh and Kerschensteiner, 2013). Below we provide a conceptual description of our model network.

In our model, wave propagation happens within the ON mosaic layer, and AC/OFF layers serve as readout. We define the connectivity and activation rules among the layers as follows: (1) ON RGCs are reciprocally coupled with nearby active ON RGCs within the dendritic interaction radius, $R_{ON}$, and becomes active for 1 s when the summed input passes some threshold. (2) ACs receive input from nearby ON cells within $R_{ON}$ and become active when the input passes a threshold. (3) Then, ACs provide inhibitory input to OFF RGCs within the dendritic radius, $R_{AC}$. An OFF RGC becomes inhibited with AC input and becomes active for 1 s upon reduction of such inhibitory input. The described connectivity is modeled as follows:

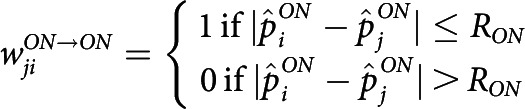


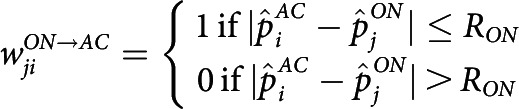


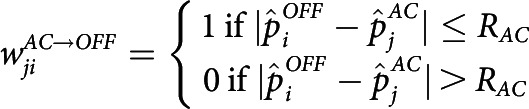
 We chose the dendritic radius of ON RGC and AC considering the size of the cell dendritic arbors experimentally measured by Akrouh and Kerschensteiner (2013), but with some flexibility regarding the effect of diffuse neurotransmitters. Parameter details are provided in Table 1.

**Table 1.** Parameters used in model simulation

Parameters	Letter	Value
Retinal wave		
Retinal wave evolution time step	Δt	100 ms
ON RGC dendritic radius	$R_{ON}$	400 μm
AC dendritic radius	$R_{AC}$	40 μm
ON RGC bursting threshold	$\Theta^{ON}$	14 unit
AII AC activation threshold	$\Theta^{AC}$	0.5 unit
OFF RGC inhibition threshold	$\Theta^{OFF}$	−0.2 unit
Wave filtering width	$\sigma_{wave}$	$0.85d_{OFF}$
RGC-V1 learning		
Retina-V1 size ratio	γ	1.98 (monkey) (Jang et al.,2020) 0.75 (cat) (Jang et al., 2020) 0.20 (mouse) (Jang et al.,2020)
Spatial decay parameter of RGC to V1connection	$d_{FF}$	18 μm (cat mosaics) 24 μm (monkey mosaics) 18 μm (mouse mosaics)
Initial weight of RGC to V1 connection	$W_{init}^{FF}$	0.05
Nonlinearity of V1 sigmoidal response curve	$\Theta_{V1}$	0.5
Nonlinearity of V1 sigmoidal response curve	$\delta_{V1}$	0.15
Time constant of firing rate average	$\tau^{FF}$	15 learning steps
Learning rate in Hebbian learning	$\epsilon^{FF}$	0.005
Learning epochs	---	15 epochs
Resource limit of single connection	$W_{limit}^{FF}$	0.14
Image filter size	$\sigma_{img}$	36 μm (cat mosaics) 56 μm (monkey mosaics)
V1 horizontal connection network learning		
Initial weight of V1 horizontal connection	$W_{init}^{V1}$	0.01
Time constant of firing rate average	$\tau^{V1}$	10 learning steps
Learning rate in Hebbian learning	$\epsilon^{V1}$	2 × 10^−7^ (cat/monkeymodels) 2 × 10^−5^ (mouse model)
Learning epochs	---	30 epochs (cat/monkeymodels) 10 epochs (mouse model)
Resource limit of single connection	$W_{limit}^{V1}$	5 × 10^−4^
V1 spontaneous activity		
Normalization weight of V1 horizontalconnection	$W_{final}^{V1}$	3
Amplitude of local random stimulus	$I_{local}$	10
Spatial scale of local random stimulus	$\sigma_{local}$	20 μm
Amplitude of background noise stimulus	$I_{background}$	0.01
Spatial scale of background noise stimulus	$\sigma_{background}$	30 μm

##### Propagation mechanism of retinal waves

We designed our model based on a cellular automaton, in which each cell (at a specific time) is assigned a discrete cell state, and the cell states at time t+Δt are determined by updating the cell states at time *t* according to a set of cell type-specific rules. We describe the cell type-specific states, input rules, and state update rules of our model below (Butts et al., 1999).

The state of an ON RGC $S^{ON}\left(t\right)$ at a given time t can be waiting, active, or inactive. If an ON RGC is in a waiting state, it can switch to an active state at the next time step by receiving input exceeding the threshold $\Theta^{ON}$ from nearby coupled ON RGCs in an active state. For realistic simulation, the amount of $\eta_{c}$, which is the input that an active cell provides to other coupled cells, is drawn from a normal distribution of mean 1 and SD ΔC=0.2. After an ON RGC becomes active, it remains active for $T_{a}=1s$, after which it becomes inactive for the rest of the simulation.

The state of an AC $S^{AC}\left(t\right)$ can be either waiting or active at a given time. When a waiting AC receives input exceeding threshold $\Theta^{AC}$ from connected ON RGCs, it becomes active in the next time step. Different from RGCs, an AC switches back to waiting state whenever it receives input that does not exceed $\Theta^{AC}$, thereby retaining the ability to become active again.

The state of an OFF RGC $S^{OFF}\left(t\right)$ can be waiting, inhibited, active, or inactive at a given time. When a waiting OFF RGC receives inhibitory (negative sign) input not exceeding the threshold, $\Theta^{OFF}$, from connected ACs, it becomes inhibited in the next time step. When inhibitory input declines and allows the threshold to be exceeded, the OFF RGC state rebounds to active, which it maintains for $T_{a}=1s$ before going inactive for the rest of the simulation. The parameter details are listed in Table 1.

##### Wave initiation, control, and postprocessing

For waveform and dynamics control, we initially assigned waiting state to only a portion (80%) of randomly selected ON RGCs and assigned inactive state to the remainder (20%) (Feller et al., 1997). We then initiated a wave at t=0 near the boundary of the extended mosaic by assigning active state to waiting ON RGCs within a circular region of radius $R_{init}=400µm$. The wave was subsequently allowed to propagate freely, while indices of active ON/OFF RGCs were recorded over time. For each mosaic, we simulated a set of 500-1000 retinal waves, each with different initial conditions and wave initiation positions.

Because the cells initially assigned inactive status cannot participate in the wave until the end (leaving “holes” in the propagating waves), we conducted a postprocessing process that ensured that all RGCs inside a propagating wave were densely active. At each time step, we spatially smoothed active state profiles of ON and OFF RGC layers using a 2D Gaussian filter of width $\sigma_{wave}=0.85d_{OFF}$. Then, we normalized the resulting activation values so that the maximum activation of each RGC layer was always 1 over time.

Finally, to ensure a uniform direction distribution, we classified the waves into 12 directional categories and sampled an equal number of waves from each category to construct a final, direction-unbiased wave dataset. From that, we imported random waves when needed.

Apart from patterned waves, we constructed a random retinal activity, permuted version of retinal waves as a control case for our horizontal network developmental model. The permutation was done simply by shuffling the RGC activation values over the RGC indices, thus preserving the overall activation level while removing correlated wave patterns.

##### Initialization of the RGC-V1 statistical wiring model

At the initial time of development, we assumed that RGCs are retinotopically wired to cortical neurons in the corresponding V1 space (Paik and Ringach, 2011; Song et al., 2018), on the basis of the retinocortical mapping ratio reported in previous experimental observations (Jang et al., 2020). To determine cortical sampling locations, we looked for every pair of data ON/OFF RGCs with a distance less than $1.5d_{OFF}$ and set their center locations as cortical sampling sites. We did not consider padded RGCs in the extended mosaic as follows:

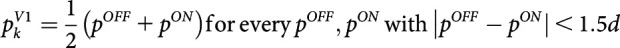


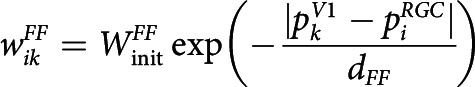


Here, $w_{ik}^{FF}$ represents the feedforward connection weight from the $i^{th}$ RGC to $k^{th}$ cortical site, where $W_{init}^{FF}$ is the initial connection weight (Sailamul et al., 2017). Parameter details are provided in Table 1.

##### Nonlinear response curve of V1 neurons

The response curve of V1 neurons was modeled as a nonlinear sigmoid kernel with parameters $\delta_{V1}$ and $\Theta_{V1}$ as follows:

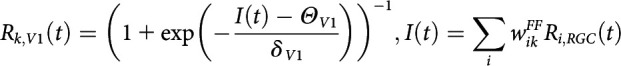
 Here, $R_{k,V1}\left(t\right)$ is the response of the $k^{th}$ cortical neuron at wave time t, where activation of $i^{th}$ RGC by the retinal wave is given by $R_{i,RGC}\left(t\right)$. Input to a cortical cell is solely determined by retinal feedforward input here, but horizontal input or direct cortical stimulus is allowed in later simulations. Parameter details are provided in Table 1.

##### V1 receptive field formation by retinal waves

After initialization with exponential pooling function, each ON/OFF subregion of the V1 “receptive field” is contributed by ∼1 RGC. To enlarge further the V1 receptive field before simulating the horizontal connection network, we followed the V1 receptive field developmental model of Song et al. (2018) to develop further the feedforward connections between the RGCs and V1 neurons. For each feedforward connection, a weight update was done once per retinal wave, following the rule below.

For a given V1 neuron receiving input from a retinal wave, we sampled the peak response of the V1 neuron and related RGC activity levels at the same time. Then, we used profiles of the sampled responses from many retinal waves for an update of the covariance rule-based weights (Sejnowski, 1977), following the formulation below.

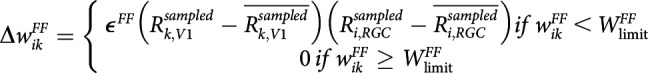


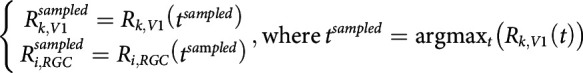


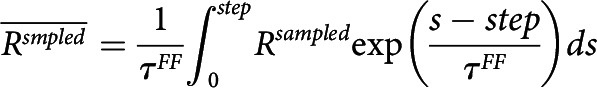
 Here, we define the learning threshold $\bar{R^{peak}}$ as the running average of the sampled responses of a cell during the learning steps. The term $\tau^{FF}$ represents how fast the threshold changes during the learning steps, and the term $\epsilon^{FF}$, the learning rate, denotes how quickly the weight update is done. We assumed that the resource for a single connection is limited by $W_{limit}^{FF}$.

We simulated the development of RGC-V1 feedforward connections using a postprocessed retinal wave dataset, 15 epochs in total. At each epoch, we shuffled wave order and iterated over all waves one by 1, applying the learning rule. All the parameter details are provided in Table 1.

##### Initialization of the V1 horizontal network model

After the RGC-V1 feedforward development was complete, we froze the feedforward connection weights and simulated development of the V1 horizontal connection network by retinal waves. Initially, we horizontally wired V1 cells with random weights as follows:

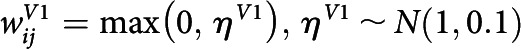
 Here, $w_{ij}^{V1}$ represents the synaptic connection weight from the $i^{th}$ to $j^{th}$ cortical site $\left(i\neq j\right)$, and $\eta^{V1}$ is drawn randomly from a normal distribution $N\left(1,0.1\right)$. After setting the random connections, we normalized each V1 cell's outgoing connection weight sum by $W_{init}^{V1}$ and finished the initialization step. Parameter details are provided in Table 1.

##### V1 horizontal network development by retinal waves

For each V1 horizontal connection, a weight update was done once per retinal wave. For a given retinal wave, responses of the V1 neurons over time were determined by the sum of feedforward input from RGCs and horizontal input from other V1 neurons as follows:

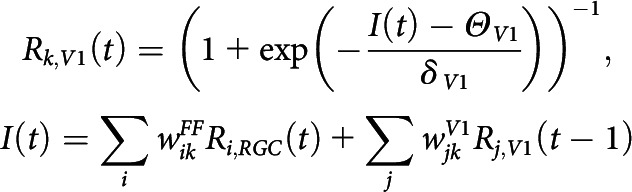
 Then, peak responses of V1 neurons were sampled and used as a response profile for a covariance rule-based weight update of the V1 horizontal network as follows:

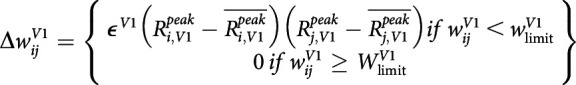


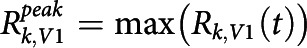


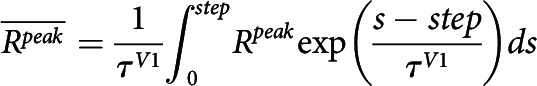
 Here, we define the learning threshold $\bar{R^{peak}}$ as the running average of peak responses of a V1 neuron over learning steps. Here, $\tau^{V1}$ represents how fast the threshold changes during the learning steps, and $\epsilon^{V1}$, the learning rate, denotes how quickly the weight update is done. We assumed that resource for a single connection is limited by $W_{limit}^{V1}$ as follows:

We simulated development of the horizontal connection network using a postprocessed retinal wave dataset (permuted dataset as control case), 30 epochs in total; at each epoch, we shuffled the wave order and iterated all the waves one by one, applying the learning rule. All the parameter details are provided in Table 1.

##### V1 horizontal network-driven spontaneous activity

After horizontal connection development was complete, we froze the horizontal connection weight and simulated spontaneous patterned activity induced by horizontal connectivity. We normalized each V1 cell's incoming connection weight sum to $W_{final}^{V1}$ and modeled driving input $I_{stim}$ for V1 network as a sum of local stimulus and global background noise, denoted by the following:

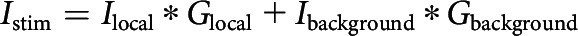
 where $G_{local}$ is the local dominant 2D Gaussian input of width of $\sigma_{local}$ given at a random location in the cortical space with peak value 1, and $G_{background}$ is background random noise drawn at each cortical neuron from $U\left(0\text{,}1\right)$, smoothed by 2D Gaussian filter of a width of $\sigma_{background}$, and normalized to have a maximum value of 1. The terms $I_{local}$ and $I_{background}$ denote the intensities of local stimulus and global background noise.

Then, in the absence of feedforward input (all $w^{FF}=0$), we provided driving input, $I_{stim}$, to neurons in the V1 horizontal network and integrated their responses recurrently until the network activity diverged as follows:

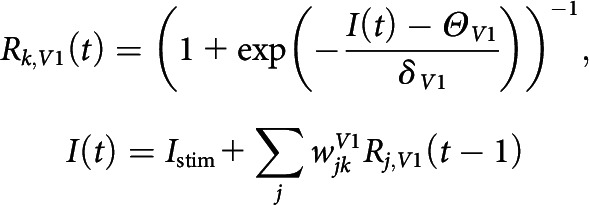


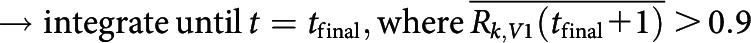
 We then spatially filtered the cortical response profile of V1 neurons using a 2D Gaussian filter of width, $\sigma_{img}$, to obtain a response image $A\left(x\right)$ where x denotes pixel position. Next, we normalized the image pixel intensities to be zero-centered and to have an SD of 1. Repeating the entire procedure, we modeled N=200 spontaneous activity images, each indexed as $A_{i}\left(x\right)$. Parameter details are provided in Table 1.

##### V1 spontaneous correlation patterns

Using the simulated spontaneous activity images $A_{i}\left(x\right)$, we computed spontaneous correlation patterns as the pairwise Pearson's correlation between activity at a reference pixel s and activity at all other pixels x, given by the following:

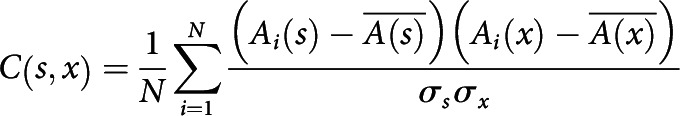
 Here, the horizontal bar denotes averaging over all spontaneous activity images, and $\sigma_{x}$ denotes the SD of activity over all images at location x (Smith et al., 2018).

##### Measurement of the cortical orientation map

We calculated the preferred orientation, $\theta_{k,OP}$, at cortical site k from the angle between the center-of-mass positions of ON/OFF RGCs as follows:

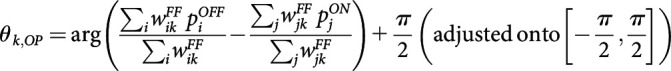
 Then, we filtered the orientation preferences of cortical neurons using a 2D Gaussian filter of width, $\sigma_{img}$, obtaining an orientation map image $OP\left(x\right)$ where x denotes pixel position. With the calculated orientation map and a given reference point s, we also computed an orientation similarity map as orientation preference similarity of all pixels x to s, given by the following:

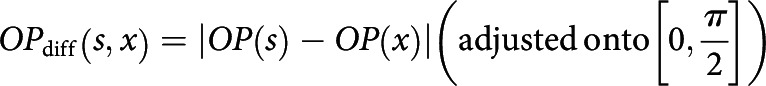


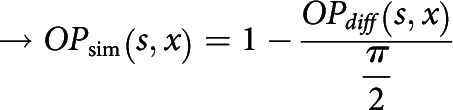
 To estimate the spatial frequency of the orientation map, the local orientation tuning was estimated at each retinal location, after which the resulting organization was smoothed by 2D Gaussian filters of different sizes to find a consistent spatial period. The peak frequency calculated from an FFT analysis of the maps was measured for various filter sizes, and the spatial frequency at a stable plateau was selected.

##### Simulation in salt-and-pepper organization

To show that patterned retinal waves can drive emergence of feature-specific connections in the salt-and-pepper organization of rodent V1, we performed additional modeling and developmental simulations of a cortical horizontal network using a mouse-data RGC mosaic (Bleckert et al., 2014).

Following the notion of Jang et al. (2020), we modeled the rodent V1 by allocating sparse cortical sampling locations over the measured RGC mosaics of a mouse (Paik and Ringach, 2011; Garrett et al., 2014; Jang et al., 2020). All other simulation settings were identical: the retinal waves were simulated over the RGC mosaic; the RGC-V1 feedforward wiring was further updated by retinal waves; and the horizontal connections were assigned and updated by retinal waves. For this model network, we only analyzed the orientation specificity of developed connections because clustering index (CI) analysis and spontaneous activity simulation assume the presence of a columnar orientation map. The simulation parameter details are provided in Table 1.

##### Simulation of synchronous Stage II retinal waves

For Stage II retinal waves, it was reported from *in vivo* experiments that a cholinergic transmission network among ACs drives synchronous wave-like responses of ON and OFF RGCs. We modeled this synchronous wave by introducing a minimally modified version of the ON/OFF asynchronous model of Stage III waves. In the modified version, the cross-inhibitory behavior of AC is suppressed, so that an ON RGC is directly coupled to nearby OFF RGCs as follows:

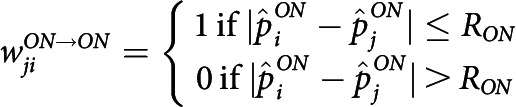


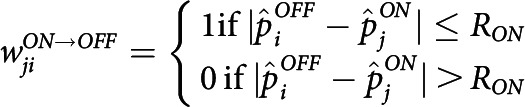
 The state of an OFF RGC $S_{OFF}(t)$ at a given time t is defined as waiting, active, or inactive. If an OFF RGC is in a waiting state, it can switch to an active state at the next time step by receiving input exceeding the modified threshold $\Theta_{OFF}=\Theta_{ON}$ from nearby coupled ON RGCs in an active state. After an OFF RGC becomes active, it remains active for $T_{a}=1s$, after which it becomes inactive for the rest of the simulation. Other than these modifications, all parameter details were the same as for the Stage III wave model.

##### Measurement of presynaptic to postsynaptic RGC-V1 activity correlation

To assess the degree of retina-V1 activity correlation during wave propagation, we measured the Pearson correlation coefficient between activity of a V1 cell and activity of an RGC connected to it. Specifically, for a given V1 cell, an ON and an OFF RGC that was very strongly connected to the given V1 cell were selected. Then, for each retinal wave, the response correlation between the V1 cell and connected ON/OFF RGC was measured around the peak timing $t_{\max}$ of response of the V1 cell. The procedure was repeated for all V1 cells for 100 waves, producing a sampled set of retina-V1 correlation coefficients.

##### Frequency modulation of retinal waves

To modulate the occurrence frequency f of waves, we changed the number of waves $\tau^{V1}$ that occurs within the averaging window, using the modulated frequency factor γ given as follows:

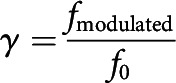


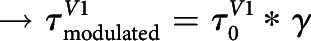
 Where $f_{0}$ and $\tau_{0}^{V1}$ represent the unmodulated values of the wave occurrence frequency and the number of waves, respectively. To simulate the condition that the occurrence of retinal waves during a unit time period increased, we modulated the frequency modulation factors $\gamma \in \left[1,2\right]$. For each γ, simulations of V1 network were repeated 5 times. The significance of changes in developmental time was tested using one-way ANOVA over different values of γ.

##### Modulation of retinal wave direction

To modulate the directional bias of wave propagation, we sampled a training set of waves biased to a specific angle, θ. In the simulation of biased waves, the waves of tilting angle within the range $\left[\theta -\frac{\pi }{12},\theta +\frac{\pi }{12}\right]$ were used. To avoid biases from the selection of θ, the above procedure was repeated 12 times for each angle $\theta =0,\frac{\pi }{6},\frac{2\pi }{6},\text{...}\frac{11\pi }{6}$. The significance of difference in connection strength was estimated using a two-sample *t* test for the average values of the LHC weight between V1 cells with orientation tuning parallel to θ and cells with orientation tuning orthogonal to θ. As a control, the procedure was done identically for a V1 network with an unbiased wave dataset.

#### Quantification and statistical analysis

##### Trend of developed V1 horizontal connection weights with respect to orientation preference

To assess how the newly developed V1 horizontal connections are related to the preferred orientations of the connected neurons, we first classified the weights of all nonzero horizontal connections into six groups according to the preferred orientation difference between the connected neuron pairs, as described below:

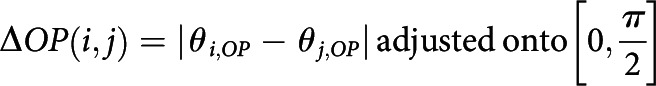


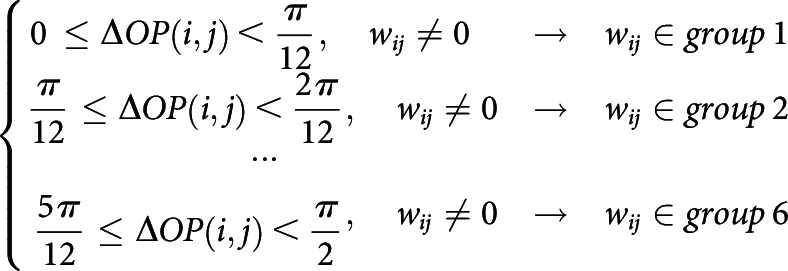
 Then, we assessed the group trends using nonparametric Cuzick's test for the trend of categorical data, under the null hypothesis that there was no trend across the groups. The *p* value was calculated from the *z* statistics given by the test. To rule out the effect of local connections, neural connections shorter than one period of the orientation map were excluded in our analysis.

For the mouse V1 model, we conducted an additional test for comparison with the experimental results of Ko et al. (2014). First, every cell pair (including pairs with zero connection weights) was categorized into one of three groups according to their orientation difference. Then, we converted all the connection weight values to Boolean coupling relations by thresholding with $\Theta_{w}=\left(1-10^{-5}\right)W_{limit}^{V1}$. From that, we tested the trend of connection probability (the number of connected cell pairs/the number of cell pairs in a group) across three groups using the Cochran-Armitage test for trend of categorical proportion, under the null hypothesis that there is no trend across the groups. The *p* value was calculated from the *z* statistics given by the test. Considering the small size of V1 in mice, all connections within the cortex were included in our analysis.

##### Spatial clustering of developed V1 horizontal connections

To quantify how much the developed cortical connections were spatially clustered in cortical domains, for a given V1 neuron, we selected the top 20% of the strongest connected postsynaptic cortical locations. Then, for the selected locations, we sought to test whether the horizontal connection network was significantly clustered. Ruthazer and Stryker (1996) used Hopkins' statistic with a sliding window to quantify clustering in the cell plots under the null hypothesis of spatial randomness. Here we summarize how we replicated their methodology. For a set of points in a given window, two basic measurements were done: (1) A 10% random subset was taken from the point set, and nearest neighbor distances from each member in the random subset to the whole set were measured (denoted w). (2) A set of random locations (with set size same as in the first measure) within the window was selected, and nearest neighbor distances from the random locations to the whole point set were measured (denoted x). For the given window, a Hopkin's statistic was computed as $\ln\left(\frac{\sum x^{2}}{\sum w^{2}}\right)$.

Given a V1 neuron and the top 20% of the strongest connected postsynaptic locations in the cortical space, we moved a circular sliding window of radius $2d_{OFF}$ and collected H-statistic values at each sliding window position. Then, we took the median value of the H-statistics over every window as the CI of the presynaptic neuron's cortical connectivity. We excluded the surrounding region of radius $2d_{OFF}$ from our analysis to check only for clustering in the remote postsynaptic area, considering the shape of the long-range connections in the V1 layer 2/3 of higher mammals.

For a developed V1 network, we obtained CI values from 50 randomly selected presynaptic neurons, following the above procedure. The same procedure was repeated for the initial random network and for the network developed from randomly permuted retinal activities as well. Moreover, the significance of long-range clustering relative to the initial network was assessed using a two-sample *t* test of CI values.

##### Similarity of developed V1 horizontal connectivity across different random initial conditions

To investigate whether horizontal networks developed from random initial conditions have a similar connectivity pattern, we repeated the developmental simulation of horizontal connections for 20 different random initial networks. We then measured the Pearson correlation coefficient between the connection weight matrices of all possible network pairs (*n* = 190). The significance of the correlation between the networks was then assessed using a *t* test for the obtained correlation values.

##### Matching between the spontaneous activity correlation map and orientation map

To quantify the alignment between the cortical activity correlation pattern and the orientation map, for a given reference point s, we measured the Pearson's correlation coefficient between the orientation similarity map, ${OP}_{sim}\left(x\right)$, and the activity correlation map, $C\left(x\right)$, of reference, s (x denotes pixel locations) as follows:

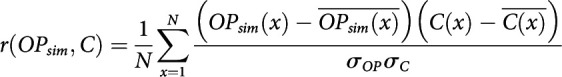
 Here, N is the number of pixels. To test for statistical significance of the spatial correlation between the two maps, we made a control version of the correlation pattern, $C^{'}\left(x\right)$, by randomly rotating the original, $C\left(x\right)$. Then, for the overlapping region, $x_{o}$, of rotated $C^{'}$ and original $OP_{sim}$, $r\left(OP_{sim}\left(x_{o}\right),C\left(x_{o}\right)\right)$ and $r\left(OP_{sim}\left(x_{o}\right),C^{'}\left(x_{o}\right)\right)$ were measured. The same analysis was done 100 times with different control maps, and the values of $r\left(OP_{sim},C\right)$ and $r\left(OP_{sim},C^{'}\right)$ were compared by paired *t* test to generate *p* value.

The above procedure assesses the significance of the correlation map and orientation map matching given a single, selected reference point. To test the global coherence of activity-orientation matching as well, we obtained $r\left(OP_{sim},C\right)$ values from all locations of the V1 neurons as reference points and tested for significance using a two-sided *t* test.

#### Data and code availability

All the data supporting the findings of this study, model simulations, and data analysis codes are available on GitHub (https://github.com/vsnnlab/rwave).

## Results

### Spontaneous retinal waves induced feature-specific long-range horizontal connections

**Figure 3.**
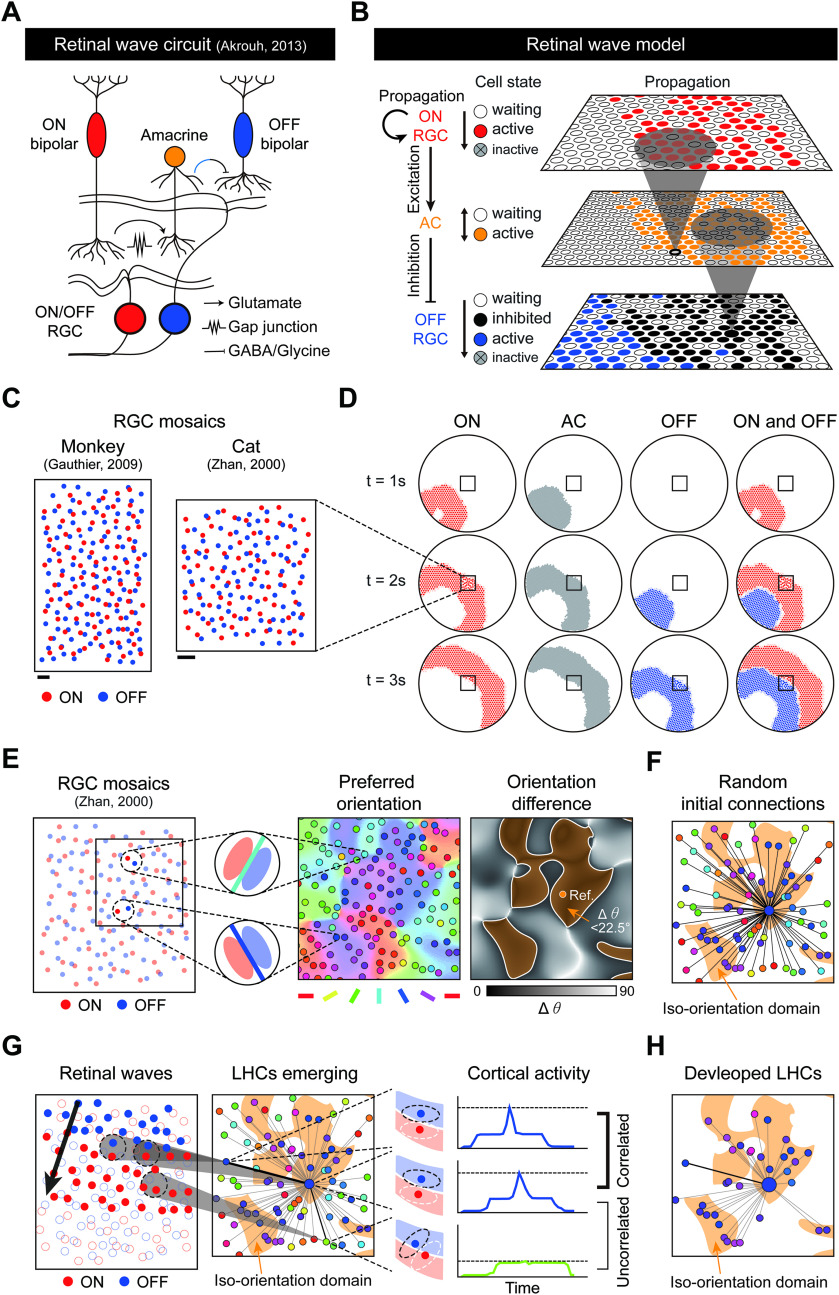
Feature-specific horizontal connections develop by retinal waves. ***A***, Simplified model of cross-inhibitory circuitry (Kerschensteiner, 2016) engaged during retinal waves. ***B***, Simplified network model that simulates the cross-inhibitory behavior of ACs and an illustration of waveform formation and propagation on overlaid hexagonal lattices of ON/OFF RGC and AC. ***C***, The wave model was simulated using data RGC mosaics measured in cats (Zhan and Troy, 2000) and monkeys (Gauthier et al., 2009). Scale bar, 100 μm. ***D***, A simulated example of propagating retinal wave for a 3 s period. Separation of ON/OFF activation regions is achieved by cross-inhibition by AC. ***E***, Left, Middle, The statistical wiring model in which orientation tuning is determined by retinal mosaics. Right, Yellow shaded area represents iso-orientation domains. ***F***, Layout of the initial horizontal connections in V1. ***G***, Simulation of the developmental model of feature-specific connectivity by retinal waves. As propagating retinal waves provide a correlated activation of cortical neurons with aligned ON/OFF receptive fields, horizontal connections between neurons with the same orientation preference are selectively enforced by the Hebbian learning rule. ***H***, Layout of the V1 horizontal connections developed by retinal waves.

We first implemented simulations of spontaneous retinal activity. We modeled retinal waves based on the propagation-readout model of the Stage II waves by Butts et al. (1999) with necessary modifications for a model of the Stage III waves. The actual retinal circuitry involving bipolar cells, diffusing glutamate and many more components, was simplified to a network containing ON/OFF RGCs and cross-inhibitory ACs (Fig. 3*A*). Based on the experimental observation that inhibitory transduction of ACs induces temporal delay between the bursting activity of ON and OFF RGCs (Akrouh and Kerschensteiner, 2013; Firl et al., 2015), our retinal wave model (Fig. 3*B*) simulates spontaneous activity as follows: an ON wavefront is formed by local excitatory networks of ON RGCs. Then local ACs are excited by surrounding ON RGCs, which cross-inhibits neighboring OFF RGCs. The OFF RGCs are activated when local inhibition declines, forming an OFF wavefront. As a result, the simulated Stage III wave at a given time appears as one of separate activities of local ON/OFF RGCs. We then simulated retinal waves using experimentally measured ON/OFF RGC cell body mosaics of a cat (Zhan and Troy, 2000) and a monkey (Gauthier et al., 2009) (Fig. 3*C*,*D*; see also Movie 1). Because the RGC mosaic data lacked information about AC locations, we made a hexagonal AC lattice from the experimentally measured density.

Movie 1. Sample retinal wave simulated on an extended mosaic of cats from Zhan and Troy (2000). 10.1523/JNEUROSCI.0649-20.2020.video.1

Next, following the statistical wiring model of the retinocortical pathway (Ringach, 2007; Paik and Ringach, 2011; Song et al., 2018), we developed a model circuit of the neurons in V1. In this model, the receptive field of each V1 neuron is developed by retinotopic inputs from local ON/OFF RGC mosaics (Fig. 3*E*). The anisotropic alignment of ON and OFF receptive fields generates the orientation preference of each V1 neuron. In the current simulation, to mimic wirings of unrefined early cortical circuits, horizontal connections between V1 neurons were added with randomly initialized synaptic strengths (Fig. 3*F*; for details, see Materials and Methods). Using this model, we simulated spontaneous generation of retinal waves and found that propagation of the ON and OFF retinal waves could provide a correlated activation of V1 neurons of similar orientation tuning (Fig. 3*G*). We confirmed that this correlated activation of V1 neurons was sufficient to strengthen their cortical wiring by a simple Hebbian plasticity implemented in horizontal connections (Fig. 3*H*).

**Figure 4.**
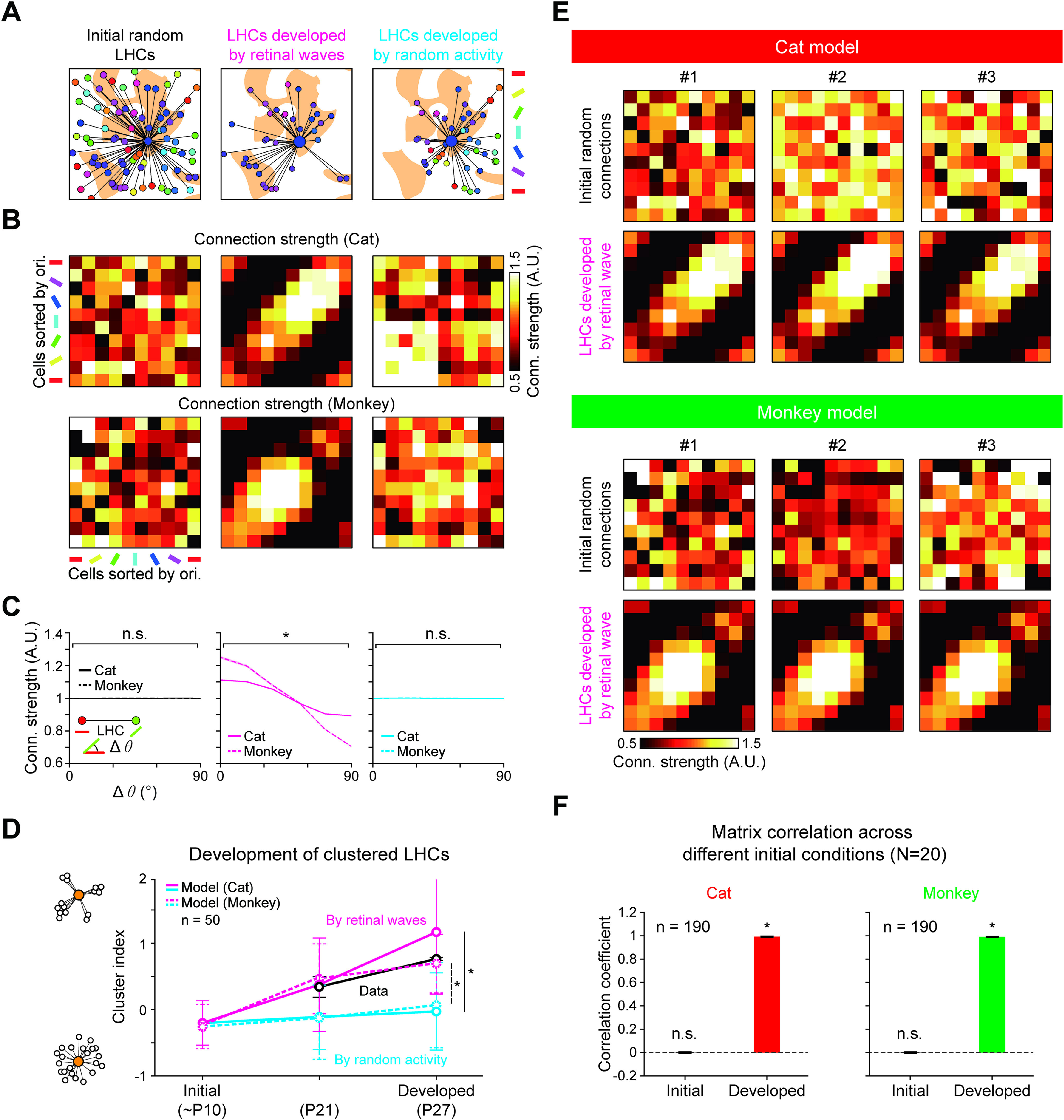
Organization of LHCs constrained by the structure of retinal afferents. ***A***, Analysis of horizontal connection strengths in the initial random LHCs (left), LHCs developed by retinal waves (middle), and LHCs developed by randomly permuted activity (right). For each network, the locations of the top 10% of the strongest postsynaptic connections for a presynaptic location are shown over the orientation difference map. ***B***, The average connection strength between neurons within the networks is summarized as connectivity matrices. Cells were batched into 10 subsets according to their orientation. Each pixel value denotes the average connection strength of LHCs between neurons in a pair of subsets. Connections of strength are normalized so that the average value in a network becomes unity. ***C***, Feature-specific LHCs developed by the model. Average connection strength was plotted as a function of orientation difference. Shaded area represents SE. ***D***, Postsynaptic clustering in developing pre-EO ferret V1 (Ruthazer and Stryker, 1996) and clustering in model V1 network developed from retinal mosaics in a cat and a monkey. Error bars indicate SD. ***E***, Repeated developmental simulations from random initial connections. The horizontal connections develop in similar forms regardless of the initial conditions. ***F***, Pairwise correlations between initial networks and between developed networks, across different initialization conditions (*N* = 20). Correlations between developed networks are close to 1, indicating convergence to a similar connectivity pattern. *p < 0.05, n.s., not significant.

After repeated propagations of spontaneously generated retinal waves in arbitrary directions, the initial random horizontal wirings turned to selective connections between neurons of similar orientation tuning (Fig. 4*A*,*B*). After training with ∼500 waves of random directions, the statistics of LHCs developed by retinal waves showed significant bias of feature specificity as observed in ferrets (Ruthazer and Stryker, 1996), whereas such biases are not observed in those developed by randomly permuted activities (Fig. 4*C*; cat, Cuzick's test for trend; *p* = 0.26, for *n* = 68,554 random initial synapses; *p* < 4.94 × 10^−327^, for *n* = 64,101 synapses developed by retinal waves; *p* = 0.11, for *n* = 68,554 synapses developed by randomly permuted retinal activities; monkey, *p* = 0.43, for *n* = 65,032 random initial synapses; *p* = 4.94 × 10^−327^, for *n* = 50,188 synapses developed by retinal waves; *p* = 0.1, for *n* = 65,032 synapses developed by randomly permuted retinal activities). We observed that the cluster indices of developed LHCs in both cat and monkey models were comparable with those observed in Ruthazer and Stryker (1996), and were significantly higher than those developed by randomly permuted activities (Fig. 4*D*; developed (cat), two-tailed paired *t* test, *n* = 50, *p = 2.43 × 10^−41^; developed (monkey), *n* = 50, *p = 5.08 × 10^−6^).

Next, to examine whether initial conditions of horizontal connectivity affect the developed structure of the feature-specific LHCs, we repeated the simulation with different initial random horizontal networks (*N* = 20). As a result, we found that the horizontal connections develop in similar forms regardless of initial conditions before development (Fig. 4*E*). To analyze this result quantitatively, we estimated the correlation among the connection strength matrix (_20_C_2_ = 190 pairs) of initial and networks developed across different initial conditions (Fig. 4*F*). We confirmed that the correlation among the connectivity matrices of developed networks was significant (cat, initial, *r* = –7.26 × 10^−5^ ± 2.23 × 10^−3^ for *n* = 190, two-tailed paired *t* test, for *n* = 190, *p* = 0.67; developed, *r* = 0.99 ± 3.46 × 10^−5^, two-tailed paired *t* test, for *n* = 190, *p* < 4.94 × 10^−327^; monkey, initial, *r* = –1.59 × 10^−4^ ± 1.46 × 10^−3^, two-tailed paired *t* test, for *n* = 190, *p* = 0.14; developed, *r* = 0.98 ± 1.95 × 10^−4^, two-tailed paired *t* test, for *n* = 190, *p* < 4.94 × 10^−327^). Notably, averaged correlation between developed networks was close to 1, indicating convergence to a similar connectivity pattern. These results suggest that the structure of feature-specific LHCs in the cortex develops under constraint by the retinal structure, regardless of the initial condition of connectivity.

### Spontaneous cortical activity induced by feature-specific long-range horizontal connections

**Figure 5.**
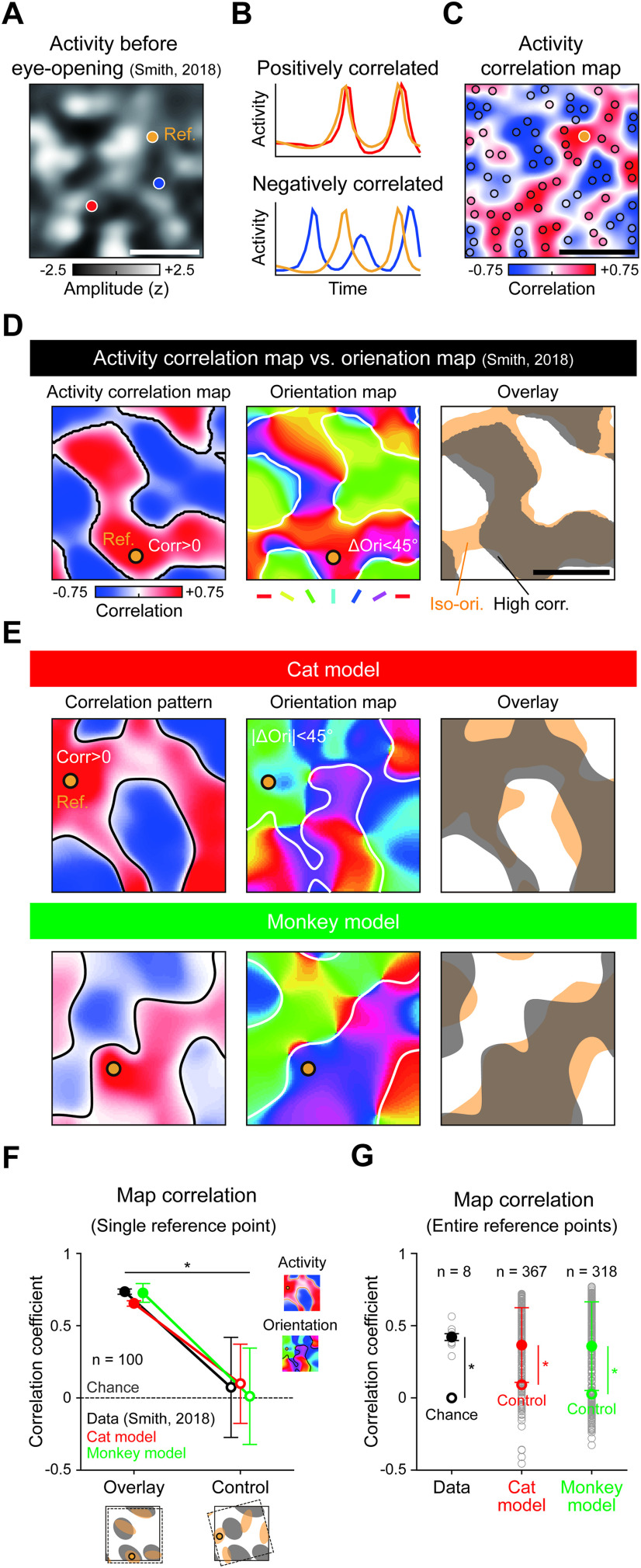
Feature-specific connectivity developed by retinal waves can underlie correlated activity in early V1. ***A***, Spontaneous events in early ferret V1 has an underlying correlation over cortical space. *z*-scored images of spontaneous events observed in ferret V1 before eye-opening. Colored points indicate cortical locations of interest (orange represents reference; red/blue represents points to be compared with reference). ***B***, Sample cortical activity correlations with respect to the reference point. Cortical activity at the red point has a higher correlation to the reference than the blue point does. ***C***, Activity correlation map underlying early V1. Pearson correlation over the entire cortical space to the reference point is computed using complete activity images. ***D***, Activity correlation map matched to orientation map in ferret V1. Left, Activity correlation map. Orange line indicates zero-correlation contour. Middle, Measured orientation map. Dark line indicates iso-orientation domain contours. Right, Alignment between positively correlated cortical regions and iso-orientation domains for a given reference point. ***E***, Activity correlation map matched to orientation map in model V1 developed by retinal mosaic in cats and monkeys. ***F***, Correlation between activity correlation map and orientation similarity map in the data and model, as tested using Pearson correlation for a given reference point. Tested map pairs in ***E***. ***G***, Correlation between activity correlation map and orientation similarity map in data and model tested for entire reference points (data: *n* = 8; cat model: *n* = 367; monkey model: *n* = 318). Data adapted with permission from Smith et al. (2018). *p < 0.05.

Next, we examined whether the developed circuits of feature-specific LHC could reproduce patterns characteristic of the spontaneous cortical activity observed in early developmental periods. It has been reported that clustered cortical activity, topographically correlated with the underlying orientation map, is observed in developing visual cortex (Chiu and Weliky, 2001; Kenet et al., 2003; Smith et al., 2018) (Fig. 5*A-C*). A recent study on female ferrets reported that the spontaneous cortical activity patterns before eye-opening predict the correlated organization of the orientation map in adults (Smith et al., 2018) (Fig. 5*D*), suggesting that spontaneous activities in V1 may initialize the topographic maps in the cortex. However, this scenario could not explain clearly how spontaneously generated cortical activity organizes into systematic columnar patterns.

Here, contrary to the above scenario that the spontaneously generated cortical activity pattern initially determines organization of the orientation map in V1, our model suggests that spontaneous retinal activity determines the patterns of both the orientation map and the activity pattern in V1, by generating horizontal wirings that connect iso-domains of the underlying orientation map. In this simple scenario, topographies of spontaneous V1 activity and underlying orientation maps must be correlated. Further, once LHCs develop, silencing the feedforward activity in a retina or LGN (Smith et al., 2018) cannot eliminate the correlated activity in V1, as observed in ferrets.

To validate this model, we removed all feedforward drive from the retina in our model and simulated activities by randomly driving the V1 network with developed LHCs. Following the analysis in a previous study (Smith et al., 2018), we selected reference points at arbitrary locations in V1 and computed the Pearson coefficient of correlation for spontaneous activities between the reference and other locations across cortical space (Fig. 5*E*, left; see also Movies 2, 3). We observed strong matching between the activity correlation map and underlying orientation map, even though the V1 circuit does not receive inputs from the feedforward pathway. As observed in ferrets (Smith et al., 2018), correlation between the activity correlation map and orientation map was significantly higher than in the controls where two maps were randomly rotated (Fig. 5*F*; two-tailed paired *t* test with randomly aligned controls; data maps: *n* = 100, *p* = 1.1 × 10^−35^, cat model: *n* = 100, *p* = 2.23 × 10^−308^; monkey model: *n* = 100, *p* = 1.90 × 10^−240^). We also repeated this for randomly chosen reference points and confirmed the statistical significance of the correlation (Fig. 5*G*; two-tailed *t* test; data maps: *n* = 8, *p* = 0.02, cat model: *n* = 367, *p* = 1.17 × 10^−41^; monkey model: *n* = 318, *p* = 4.94 × 10^−47^). These results suggest that the observed correlation between spontaneous V1 activity and orientation maps can readily be explained by our model.

Movie 2. Change of activity correlation pattern as reference point slides over cortical space. Simulation based on cell body mosaics in cats from Zhan and Troy (2000). 10.1523/JNEUROSCI.0649-20.2020.video.2

Movie 3. Change of activity correlation pattern as reference point slides over cortical space. Simulation based on cell body mosaics in monkeys from Gauthier et al. (2009) . 10.1523/JNEUROSCI.0649-20.2020.video.3

### Development of feature-specific horizontal connections without a periodic map

**Figure 6.**
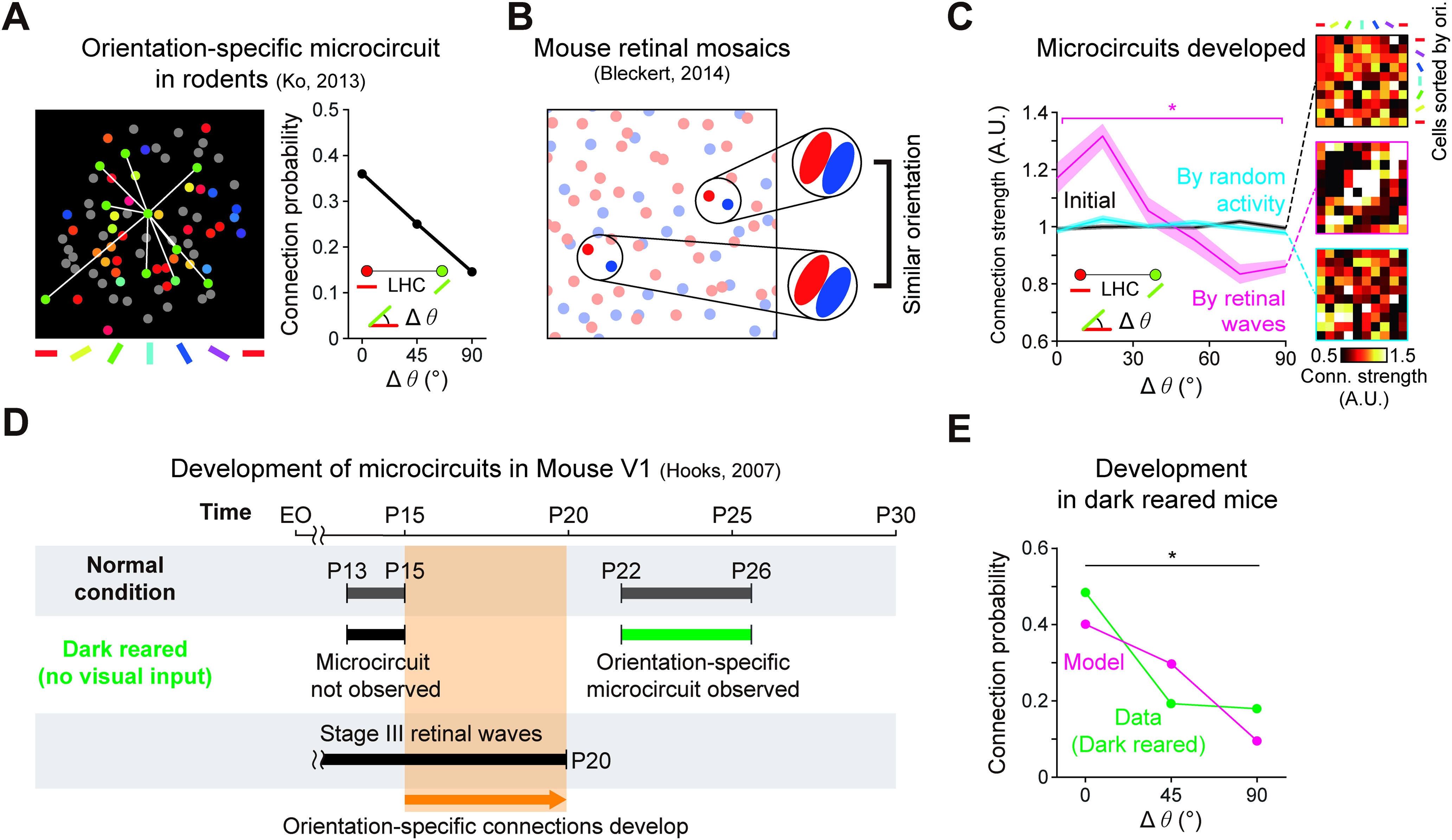
Retinal waves can drive the emergence of feature-specific cortical connections in rodent V1. ***A***, Orientation-specific microcircuits observed in mouse V1 of salt-and-pepper organization of orientation tuning (Ko et al., 2013). ***B***, Emergence of feature-specific horizontal connections by retinal waves in salt-and-pepper organization simulated with mouse retinal mosaics (Bleckert et al., 2014). The model predicts that feature-specific connections develop, regardless of the spatial distribution of orientation preference in V1. ***C***, Feature-specific microcircuits developed by a simple orientation-correlated activation model. Shaded area represents SE. ***D***, Developmental timeline of mouse V1 horizontal microcircuits. Feature-specific horizontal microcircuits emerge when the retinal wave is present and the retinocortical pathway has developed. ***E***, Feature-specific connection developed in model mouse V1 and data (Ko et al., 2014). In both model and data, the feature-specific microcircuit is observed, even if no visual experience is given during development. Data adapted with permission from Ko et al. (2014). *p < 0.05.

So far, we have shown that our model provides an explanation for how feature-specific LHCs develop spontaneously in V1 of higher mammals with columnar orientation maps. Next, we show that our model further explains how feature-specific microcircuits also develop in rodents V1 with salt-and-pepper organizations (Ko et al., 2011) (Fig. 6*A*). It is notable that the key assumption of our model is that retinal waves coactivate V1 neurons of similar tuning to develop microcircuits between them, and that this mechanism works regardless of the spatial organization of orientation preference in V1 (Fig. 6*B*).

To validate our prediction in salt-and-pepper type organizations of V1, we implemented a model V1 circuit with salt-and-pepper organization. Using mouse retinal-mosaic data (Bleckert et al., 2014) and the same developmental model for cats and monkeys, we confirmed that cortical neurons of similar orientation tuning tend to fire in correlated patterns. As a result, similar to the V1 model with a periodic orientation map, our model showed that microcircuits with significant feature specificity developed (Fig. 6*C*; Cuzick's test for trend; initial: *n* = 1122, *p* = 0.21; developed by retinal waves: *n* = 911, *p* < 4.94 × 10^−327^; developed by randomly permuted activity: *n* = 1122, *p* = 0.14). This result demonstrates that feature-specific LHCs can also emerge from the correlated activity induced by retinal waves, even in salt-and-pepper type organizations (Van Hooser, 2005), as observed in layer 2/3 horizontal microcircuits of rodent V1 (Ko et al., 2011). Notably, observations that feature-specific microcircuits appear to develop, even in dark rearing conditions with no visual experience, may also support our model. Our model predicts that these microcircuits can develop in dark rearing conditions, because spontaneous retinal waves can contribute under this condition (Hooks and Chen, 2007; Ko et al., 2014) (Fig. 6*D*,*E*; Cochran-Armitage test; model: *n* = 551, *p* = 9.87 × 10^−13^; data (dark reared): *n* = 6, *p* = 0.028). These results imply that our model can provide a universal principle for the developmental mechanism of LHCs in both higher mammals and rodents.

### Experimental predictions for retinal wave modulation

**Figure 7.**
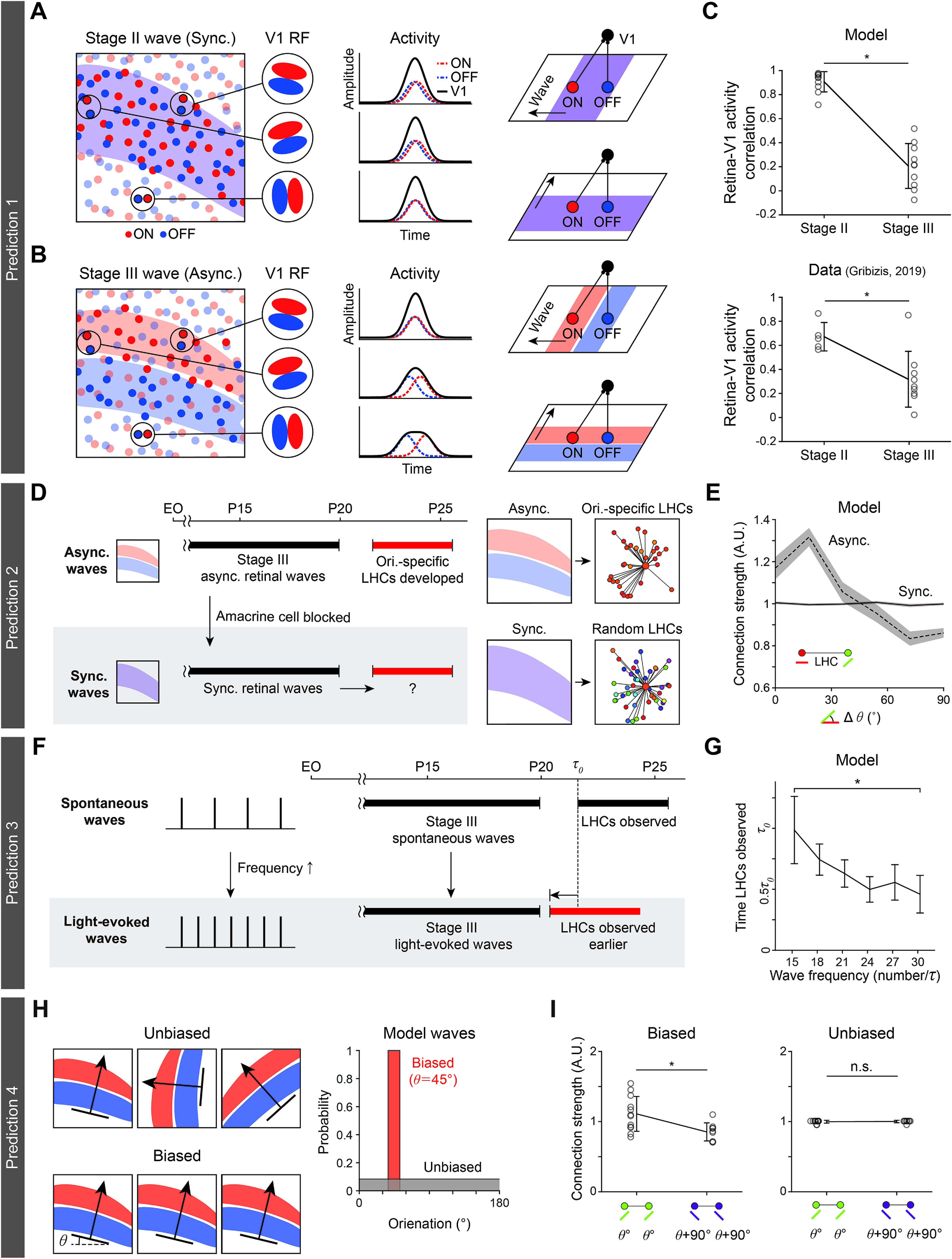
Experimental predictions for retinal wave modulation. ***A–C***, Prediction 1: Correlation between retinal activity and cortical activity will weaken during the transition of the retinal wave from Stage II to Stage III. ***A***, An ON/OFF synchronous retinal wave in Developmental Stage II. The ON and OFF retinal afferents induce V1 cell activities of similar amplitude, regardless of the spatial organization of the receptive field. This causes high average correlation between retinal and cortical activities. ***B***, An ON/OFF asynchronous retinal wave in Developmental Stage III. The ON and OFF retinal afferents induce higher V1 cell activity when the spatial organization of the receptive field matches the direction of the wave than when it is unmatched. This causes relatively lower average correlation between the retinal and cortical activities than in ***A***. ***C***, Pairwise correlation between activity of the V1 cells and the connected retinal cells, driven by Stage II and Stage III waves, respectively. The model prediction is consistent with the observation in mice V1 (Gribizis et al., 2019). Error bars indicate SD. ***D***, ***E***, Prediction 2: LHC developed by synchronous wave is not orientation-specific. ***D***, Simulated modulation of Stage III waves from asynchronous to synchronous, Stage II-like waves at P13-P15 when orientation-specific LHCs are not yet observed. ***E***, Orientation specificity of model V1 LHCs disappears because ON/OFF synchronous waves drive V1 cells independently of their orientation tuning. Shaded area represents SE. ***F***, ***G***, Prediction 3: Light-evoked wave accelerates the LHC developments. ***F***, Simulated modulation of the frequency of Stage III waves by artificial stimulation with light. ***G***, Development of orientation-specific LHCs in model V1 is expedited by increased wave frequency. Error bar indicates SD. ***H***, ***I***, Prediction 4: Light-evoked wave will bias the orientation-specific LHC connectivity. ***H***, Simulations of artificially evoked waves biased to a particular direction (θ). ***I***, The V1 model network driven by the biased waves generates more LHCs between iso-orientation domains tuned to that particular orientation than those between domains tuned to orthogonal (θ+90°) direction. Such a bias is not observed in the model V1 network driven by unbiased waves. *p < 0.05, n.s., not significant.

Our model provides experimental predictions that can be confirmed by existing data or could be validated by future studies. First, our model predicts that Stage II retinal waves (ON and OFF synchronous) and Stage III waves (asynchronous) would drive V1 cells differentially, and that the correlation between retinal activity and cortical activity would weaken during the transition of the retinal wave from Stage II to Stage III (Fig. 7*A*,*B*). The model predicts that cortical activity patterns in Stage II would be strongly correlated to retinal activity (Fig. 7*A*), because both ON and OFF waves drive V1 neurons simultaneously, regardless of the organization of the ON-OFF receptive fields of target neurons. On the other hand, in Stage III, ON and OFF waves would be asynchronous (with a noticeable time delay between them), and thus drive cortical neurons selectively depending on the spatial organization of their ON and OFF receptive fields (Fig. 7*B*). As a result, a smaller portion of the V1 neurons would be activated by instantaneous retinal waves in Stage III than in Stage II, and the correlation between the retinal and cortical activities would appear weaker.

To quantify the predicted modulation of retina-V1 correlation in the model, we performed simulations of a model network with synchronous Stage II waves achieved by silencing AC in the model, so that ON RGCs could directly drive the OFF RGCs. Using these synchronous model Stage II waves with asynchronous Stage III waves, we investigated a change in the retina-V1 activity correlation (presynaptic to postsynaptic correlations between model RGCs and connected V1 cells). We confirmed that retina-V1 correlation induced by Stage II waves is significantly reduced by the transition to Stage III waves, as previously observed in experiments of both full-field and presynaptic to postsynaptic activities (Gribizis et al., 2019) (Fig. 7*C*; two-sample *t* test; model: *p* = 4.28 × 10^−6^ for *n*_Stage II_ = 10, *n*_Stage III_ = 10; mouse data: *p* < 0.001 for *n*_Stage II_ = 5, *n*_Stage III_ = 9). These results imply that Stage II and III waves may stimulate V1 neurons in distinct ways due to their different temporal dynamics, as predicted by our model.

Next, our model suggests three additional experimental predictions for further validation of the model. That is, our model predicts that the orientation-specific LHC might not develop, or significantly weaken, if the Stage III retinal waves are suppressed or modulated in various ways (Fig. 7*D*). For example, treatment of ACs with inhibitory antagonists would revert Stage III waves to waves like those of Stage II (Gribizis et al., 2019). This may prevent the emergence of the orientation-specific LHCs or significantly weaken their connection selectivity for similar orientation tuning. We specifically proposed an experiment to manipulate Stage III waves to become waves like those in Stage II just before eye opening (∼P13) in mice, when orientation selectivity of V1 neurons is observed but LHCs are not. Our simulations show that, even if the LHCs can develop, their orientation specificity will not be observed under this condition (Fig. 7*E*; Cuzick's test for trend; Sync wave, *n* = 1336, *p* = 0.30; Async wave, mouse model: *n* = 911, *p* < 4.94 × 10^−327^).

Next, the model suggests another prediction that orientation-specific LHC might be observed earlier, if additional Stage III waves are evoked artificially. Recently, it was reported that Stage III waves could be modulated by light stimulation, which increases the frequency of those waves (Tiriac et al., 2018). Our model predicts that this would expedite the emergence of LHCs and that orientation-specific LHCs would be observed earlier than in a normal condition (Fig. 7*F*). Our quantitative analysis of the model simulation results shows that the occurrence of orientation-specific LHCs can be modulated, depending on the frequency of the artificially evoked waves (Fig. 7*G*; one-way ANOVA, *n* = 5 samples per a wave frequency; *p* = 0.0014).

Last, the model predicts that the spatial organization of LHCs would be biased to specific orientations by controlled artificial light simulations (Fig. 7*H*). Retinal waves biased to a specific angle (θ), if evoked artificially by light stimulations, would reproduce more or stronger LHCs between neurons tuned to that particular orientation. This prediction can be tested by comparing the number of LHCs connecting neurons with the preferred angle in the biased wave direction (θ) and that in the orthogonal orientation (θ + 90°). Experimentally, the number of LHCs in an orientation domain could be obtained by injection of extracellular biocytin at that local position, and then counting the number of terminal boutons (Bosking et al., 1997). Our model simulations predict a higher chance of observing LHCs between neurons with the preferred angle in the biased wave direction than that in the orthogonal orientation (Fig. 7*I*; two-tailed *t* test; biased: *n* = 12, *p* = 0.02; unbiased:*n* = 12, *p* = 0.57).

## Discussion

Results from a number of studies have suggested that retinal waves may play an important role in the development of early visual circuits, such as retinogeniculate pathways, geniculate receptive fields, and geniculocortical projections (Kaneko et al., 2005; Davis et al., 2015; Arroyo and Feller, 2016). However, before now, it was not known whether retinal waves could also contribute to the early organization of functional circuits in V1. In the current study, we demonstrated that spontaneous retinal waves can drive the development of feature-specific intracortical circuits in V1.

Regarding the initialization of LHCs in V1, the underlying mechanism of how cotuned cortical neurons fire together, even before there is visual experience, has remained unclear and needed to be investigated further (Ko et al., 2014). Our theoretical model suggests a scenario in which the development of LHCs is based on observations of the temporal asynchrony between ON/OFF activations of Stage III waves in experiments (Kerschensteiner and Wong, 2008). The idea that asynchrony of the retinal waves could contribute to the segregation of ON/OFF afferents was previously suggested by Kerschensteiner and Wong (2008) and Gjorgjieva et al. (2009), but how the segregated ON/OFF afferents contribute to the development of orientation-specific LHCs was not addressed. Extending the scenario, our model suggests that a key mechanism is that the spatial organization of the retinal mosaics can be projected onto V1 via segregated ON/OFF afferents, and this can initialize orientation-selective activation of V1 neurons, leading to the development of LHCs.

It is also possible that correlated activity among clusters of cotuned V1 neurons could be initiated by other factors, such as spontaneous activity generated by V1 neurons or the feedback input from higher visual areas. In particular, some of the previous studies suggested that spontaneous interactions within V1 could establish spontaneous modular activities, and lead to the development of LHCs (Grabska-Barwinska and von der Malsburg, 2008; Smith et al., 2018). It was proposed that local circuits in V1 might generate patterned activities spontaneously, which would serve as a scaffold for orientation columns to develop (Shouval et al., 2000; Grabska-Barwinska and von der Malsburg, 2008; Smith et al., 2018). Such cortical activity might explain how the activity of multiple clustered regions in the cortex is correlated; however, this scenario lacks explanation of a mechanism by which orientation-selective cortical receptive fields arise initially. Moreover, this model could not account for the strong and precise relationship between the retinal and cortical receptive field structures reported recently (Kremkow et al., 2016; Lee et al., 2016). Importantly, these models could not provide explanation of how LHCs could be developed in auditory cortex when retinal afferents are provided (Sharma et al., 2000). Thus, the contribution of the endogenous cortical network alone does not explain how the feature-specific horizontal connections develop and become correlated with the orientation tuning in V1.

Despite that several model studies have suggested that retinal afferents might not seed V1 orientation preference (Hore et al., 2012; Schottdorf et al., 2014, 2015), evidence from experimental studies indicates that the orientation tuning of V1 neurons originates from the local ON and OFF feedforward afferents (Jin et al., 2011; Kremkow et al., 2016). This provides a possible scenario for the development of both the initial layout of the orientation map and the organization of LHCs between iso-orientation domains. Our model suggests that the organization of ON and OFF retinal mosaics provides a blueprint for the development of the orientation map and clustered LHCs, which was not solely explained by the spontaneous activation in V1. It is also notable that the results from another experimental study support the primary role of the retinal activity in the development of LHCs. A study by Sharma et al. (2000) showed that orientation-specific LHCs can develop in the primary auditory cortex (A1), when the retinal inputs are wired to A1, whereas LHCs are rarely observed in a normal condition. Overall, these results imply that retinal activity is the strongest candidate as the source of the activity that drives the development of LHCs. This retinal origin model explains the emergence of correlated activity patterns in V1, topographic matching to the orientation map, and how these processes are performed before the onset of any visual experience. Our results suggest a simple, but powerful, model of the developmental mechanism underlying the origin of spontaneous activity patterns in V1, and its correlation to the orientation tuning maps to complete the scenario.

Observations that support our developmental model were also reported in previous studies. Durack et al. (1996) found that initial clustering of LHCs in ferret V1 coincides with, but does not precede, the development of orientation preference. This implies that the development of LHCs may “reflect,” rather than “seed,” the structure of orientation maps. In addition, feature-specific microcircuits of V1 in mice appear to emerge after development of retinocortical projections and orientation tuning of V1 neurons (Ko et al., 2013). These results also suggest that, after the feedforward pathway has been developed to induce cortical orientation tuning, retinal waves drive the development of feature-specific horizontal connections.

The previous study reported that clustered horizontal connections are observed even after binocular enucleation (Ruthazer and Stryker, 1996). However, this result cannot invalidate the role of the patterned retinal activity for cortical development of LHCs. It should be noted that the enucleation of the retinae was performed late in development (P21), after early orientation tuning and related spatial organization were already established in V1. At that time, feature-specific LHCs are expected to exist already, and might be refined by cortical activities (Sharma et al., 2000). Furthermore, another study reported a counterexample that the clustering of LHCs was not observed in cats with earlier binocular deprivation in the developmental stage (Callaway and Katz, 1991). These results are readily explained by our model: Once orientation tuning and LHCs in V1 are established by early retinal afferents (probably early Stage III), they develop without further contribution from the feedforward retinal activity in later stages.

Consistent with previous observations, our model proposes that Stage II and III retinal waves may have distinct roles in development of LHCs. That is, the Stage II retinal activity first induces development of the retinogeniculate and geniculocortical pathways (Ackman et al., 2012; Kirkby et al., 2013); then the Stage III retinal activity drives the development of LHCs (Davis et al., 2015) until eye-opening (Liets et al., 2003; Akrouh and Kerschensteiner, 2013). Another recent study on the Stage II and III retinal activities in mice also provides supporting evidence for our model (Gribizis et al., 2019). In this paper, it was reported that the global correlation between cortical activity across the cortical surface and the presynaptic retinal activity is significantly higher during Stage II than during Stage III. These results are consistent with the scenario that our model predicts: (1) Retinal activity during the Stage II period develops retinocortical pathways with a retinotopic organization and activates all the cortical neurons. This generates a global correlation of cortical activity. (2) Then, asynchronous retinal waves during Stage III selectively activate cortical neurons of similar orientation tuning by propagating in the direction of the wave. This leads to relatively lower global correlation than in the previous stage. Together, these results imply that Stage II and III retinal activities stimulate the cortical neurons in a distinct way and contribute differently to the development of LHCs.

There exist conflicting results on whether the ON/OFF asynchrony of Stage III retinal waves is significant in higher mammals. Wong et al. (1996) and Lee et al. (2002) reported that there was no noticeable temporal delay between ON and OFF RGC activity in ferret Stage III waves, whereas Liets et al. (2003) reported the existence of clear asynchrony up to ∼1 s, similar to the ON/OFF asynchrony in mouse. In the case of ferrets, it is possible that asynchrony of activity between RGC types might originate from a different mechanism, such as dendrite morphology or other neuronal dynamics, which eventually works similar to ON/OFF asynchrony in mice. Future studies are needed to observe detailed dynamics for retinal waves to drive cortical organization in each species of various structures in feedforward projections.

Our model explains how visual cortical networks develop before experience, but experimental results suggest that sensory experience is essential for the maintenance and maturation of cortical network structures. Immediately after eye-opening, an explosive increase in the synaptic density of cortical layer 2/3 (including LHCs) is observed. Ferrets dark-reared at this developmental stage appear to have much weaker orientation tuning, and their LHC clusters do not form properly (White and Fitzpatrick, 2007). Similar observations were reported in rodent V1 as well; although feature-specific cortical microcircuits are observed to develop in dark-rearing environments, they require visual experience for appropriate pruning and maturation later (Ko et al., 2014). These observations suggest that early feedforward projections are able to guide organization of the functional circuits in the cortex initially, but also that visual experience is required to drive further development of circuits for complete visual function in adult animals.

In conclusion, our results suggest that the structure of retinal mosaics and the spontaneous wave activity from them can induce early tuning maps and the feature-specific LHC circuits in V1. Our model provides further understanding of how functional architectures in the cortex can originate from the spatial organization of the periphery, without sensory inputs during early developmental periods.
